# Circadian Rhythm Modulates the Therapeutic Activity of Pulsed Electromagnetic Fields on Intervertebral Disc Degeneration in Rats

**DOI:** 10.1155/2022/9067611

**Published:** 2022-03-25

**Authors:** Yi Zheng, Yiming Hao, Bing Xia, Liangwei Mei, Shengyou Li, Xue Gao, Teng Ma, Bin Wei, Zhifen Tan, Pingheng Lan, Zhuojing Luo, Da Jing, Jinghui Huang

**Affiliations:** ^1^Department of Orthopedics, Xijing Hospital, Fourth Military Medical University, Xi'an, China 710032; ^2^Faculty of Life Sciences, Northwest University, Xi'an, China 710069; ^3^College of Medical Technology, Shaanxi University of Chinese Medicine, Xianyang, China 712046; ^4^Department of Orthopedics, The First Affiliated Hospital of Xiamen University, Xiamen University, Fujian, China 361102; ^5^Department of Biomedical Engineering, Fourth Military Medical University, Xi'an, China 710032

## Abstract

Circadian rhythm (CR) imparts significant benefits in treating multiple diseases, such as heart diseases and arthritis. But the CR effect on intervertebral disc degeneration (IVDD) therapy remains unclear. Recent studies revealed that pulsed electromagnetic fields (PEMF) are capable of alleviating IVDD. In this study, we evaluated the CR-mediated regulation of PEMF therapeutic effect on IVDD induced by rat tail disc needle puncture. Our results demonstrated that the daytime PEMF stimulation (DPEMF) is more effective than the nighttime PEMF (NPEMF) in delaying IVDD. Moreover, the rats treated with DPEMF maintained better disc stability and histology after 8 weeks, relative to NPEMF. CR and PEMF cotherapies were also examined in cellular models, whereby serum shock was used to induce different levels of clock gene expression in the nucleus pulposus (NP), thus imitating CR *in vitro*. PEMF at ZT8 (higher level of clock gene expression) correlated with a higher extracellular matrix (ECM) component expression, compared to ZT20 (lower level of clock gene expression). Taken together, these data suggest a strong role of CR in regulating the beneficial effect of PEMF on IVDD. Our findings provide a potential clinical significance of CR in optimizing PEMF positive effects on IVDD.

## 1. Introduction

Low back pain (LBP) and subsequent disability are major global concerns [1, 2]. Approximately 80% of individuals experience LBP in their lifetime, and 4-33% of people suffer from it throughout their lives, thereby resulting in enormous social and economic burdens for the community and individual patient [3, 4]. Intervertebral disc degeneration (IVDD) is the most common cause of LBP. The intervertebral disc (IVD) consists of a single gel-like nucleus pulposus (NP) and fibrocartilaginous annulus fibrosus (AF), which is sandwiched between the upper and lower cartilage endplates (EP) [5]. NP makes up the core component of IVD, which includes NP cells and extracellular matrix (ECM) components, namely, proteoglycan, collagen I, and collagen II. Inflammation, oxidative stress, and other factors cause NP dysfunction and disrupt balance between ECM synthesis and decomposition, which, in turn, accelerate IVDD and produce discogenic back pain. Unfortunately, conventional treatments such as drugs and surgical interventions cannot reverse this process [6, 7]. Therefore, noninvasive treatments are gaining attention in promoting disc regeneration.

Pulsed electromagnetic field (PEMF), a noninvasive biophysical stimulus, is reported to possess ample therapeutic effects on certain skeletal diseases, such as osteoporosis, osteoarthritis, and spinal fusion [8–10]. However, PEMF treatment for IVDD is still in its infancy. Recent studies demonstrated that PEMF has the potential to treat discogenic back pain, secondary to IVDD [11–14]. Recent reports revealed that PEMF not only reduced IVDD-associated gene expression *in vitro* but also suppressed acute inflammatory cytokine expression in a 7-day treatment of IVDD in a rat-tail needle-puncture model [11–14]. Thus far, long-term effects of PEMF stimulation on IVDD histomorphological and radiographic changes are unknown. The therapeutic effects of PEMF are known to depend on multiple factors, including frequency, duration, and amplitude [15–17]. However, other factors also affect PEMF efficacy in the treatment of IVDD and these factors remain an area of ongoing research.

Circadian rhythm (CR) is a cyclic endogenous biological pattern crucial for maintaining the 24-hour periodic cycle of physiological and behavioral alterations [18, 19]. The concept of chronotherapy, defined as the administration of treatments at appropriate time points, according to the body's biological rhythms, gained extensive attention due to its maximum efficacy and reduced toxicity of medical treatments [20, 21]. The chronotherapeutic principles have been successfully applied in cardiovascular and cancer treatments [22–24]. Recent studies revealed that the IVD physiology is strongly affected by the biological rhythm related to the activity/rest cycle, i.e., mechanical stimulation during the day and rest at night for repair [25, 26]. In addition, circadian gene oscillations were recently verified in IVD tissue [27–29]. Given these evidences, CR may be crucial in the treatment of IVDD. However, to our best knowledge, chronotherapy has not been applied in IVDD treatment. Our prior studies revealed that PEMF efficacy is affected by CR during osteoporosis treatment and during peripheral nerve regeneration [30, 31]. However, it is still unknown whether CR serves a regulatory function in the PEMF-based IVDD therapy.

## 2. Method

### 2.1. PEMF Stimulators

PEMF stimulators were obtained from GHY-III, Air Force Military Medical University (AMMU), Xi'an, China; China Patent no. ZL02224739.4 [32, 33]. To conduct animal experiments, we used a PEMF generator and three Helmholtz coils of 800 mm diameters. Specifically, the coils were located coaxially with an interval of 304 mm, and each coil consisted of enameled coated copper wire, with a diameter of 0.8 mm. The PEMF waveform comprised of a pulsed burst (burst width 5 ms; pulse width, 0.2 ms; pulse wait, 0.02 ms; burst wait, 60 ms; pulse rise and fall times: 0.3 *μ*s, 2.0 *μ*s) repeated at 15 Hz. This particular waveform was shown to promote nerve and bone regeneration in previous studies and was similar to the setting of PEMF in treating IVDD [30, 31, 34]. An oscilloscope (6000 series; Agilent Technologies, USA) was used to visualize wave shape and frequency and a Gaussmeter (Model 455 DSP; Lake Shore Cryotronics) for monitoring the accuracy of PEMF output. The rats in the treatment groups received PEMF stimulation 4 hours per day immediately after surgery. In case of cellular experiments, different Helmholtz coils were used, which was composed of two coils at a 100 mm interval distance.

### 2.2. Animal, Operational, and Experimental Protocols

All animal experiments followed the animal care guidelines approved by the Animal Research Committee of the Fourth Military Medical University. Twenty, 3-month-old male Sprague-Dawley rats were selected (weight: 260-280 g, from Laboratory Animal Center of FMMU). Water and food were given ad libitum. The temperature was maintained at 24 ± 2°C, with 50–60% humidity. The light schedule included 12 hours of daylight from 7: 00 AM and 12 hours of darkness from 7:00 PM. The rats were conditioned to the laboratory and observation conditions ≥ 30 min a day for 7 days prior to experimentation. All rats were anesthetized intraperitoneally with pentobarbital solution (1%, 40 mg/kg). Rats were arbitrarily separated into 4 groups: sham (*n* = 5); needle puncture (*n* = 5); needle puncture with daytime (7:00-11:00) PEMF treatment, DPEMF (*n* = 5); and needle puncture with nighttime (19:00-2300) PEMF treatment, NPEMF (*n* = 5). As previous studies described [35, 36], after disinfecting the tail skin with iodophor and alcohol, a 20-gauge needle was utilized to fully puncture tail disc from Co6 to Co10 levels; the needle was rotated 180 and left for 60 seconds before removal. After the operation, iodophor was used to disinfect the stab site. In the sham group (*n* = 5), the rats were placed under anesthesia without needle puncture injuries. Following the operation, all the animals were brought back to their cages and given food and water ad libitum. The rats in the experimental group were exposed to PEMF treatment (4 h/d) immediately after surgery. According to previous studies, after 8 weeks of treatment, rats were sacrificed and tested immediately [35, 36]. Before specimen harvest, rats were sent to take X-ray and MRI for the disc height and IVDD grade analysis. The Co6/Co7 discs, including endplate and bone, were excised and fixed in 4% paraformaldehyde for histological staining. The Co7/Co8 and Co8/Co9 discs were collected and instantly frozen in liquid nitrogen before storage at -80°C for transcriptome sequencing (mRNA-seq) and real-time RT-PCR. The Co9/Co10 disc was excised and maintained at -80°C for subsequent analysis.

### 2.3. Rat Primary NP Cell Culture and Treatment

10 male Sprague-Dawley rats (3-4 weeks old) were euthanized with an overdose of 1% sodium pentobarbital solution and NP cells were isolated, according to prior reports [37, 38]. In brief, the rat tail was removed under aseptic conditions, NP tissues were collected under a stereo microscope, digested in 0.1% type II collagenase for 4 hours at 37°C, centrifuged at 1000 rpm for 5 minutes, the supernatant was removed, and the pellet was resuspended in DMEM/F12 (1 : 1) medium (with 10% FBS, 1% P/S). Finally, the suspension was transferred to a culture flask and cultured in an incubator containing 5% CO_2_ at 37°C. Cells from passage 2 to 4 were used for subsequent experimentation. In the first set of the *in vitro* studies, NP cells were exposed to PEMF for 4 hours, and cellular toxicity was assessed via flow cytometry at four separate times (0 h, 24 h, 48 h, and 72 h). In the second part of *in vitro* studies, cells received serum shock to induce expression of clock genes, as previously reported [39–41]. In brief, cells were starved for 48 h before stimulation with serum-rich medium (50% DMEM/F12 and 50% fetal bovine serum) for an additional 2 h. Subsequently, following DMEM/F12 (without serum) wash, cells were grown in serum-free DMEM/F12. NP cells were then harvested after 4 h, 8 h, 12 h, 16 h, 20 h, 24 h, 28 h, 32 h, 36 h, 40 h, 44 h, and 48 h from the initiation of serum shock. To induce PEMF stimulation, NP cells were treated with PEMF at time points 8 h and 20 h, respectively, for 4 h. After 24 h incubation, the cells were collected for molecular expression evaluation.

### 2.4. Radiographic and MRI Analyses

Rat X-ray and MRI images were obtained 8 weeks posttreatment. X-ray imaging of rat tail discs was performed using an X-ray system (Faxitron Bioptics, LLC, Wheeling, IL, USA). The disc height index (DHI) was computed by two blinded spinal surgeons, as described previously [42]. Alterations in DHI was represented by % DHI, which were standardized with preoperative DHI (%DHI = 100 × postoperative DHI/preoperative DHI). MRI images were performed on an MRI system (Siemens 3T Magnetom Trio Tim scanner, Munich, Germany). The IVDD score was also calculated by two spinal surgeons, based on the Pfirrmann grading system, ranging from grade I to grade IV [43].

### 2.5. Micro-CT

Following 8 weeks of treatment, rats were sacrificed, and subsequently, the tail vertebrae (Co6-Co7) were harvested and fixed in 4% paraformaldehyde. Prior to histological processing, samples were scanned with micro-CT (eXplore Locus SP; GE, Fairfield, US) using X-ray tube settings of 80 kV and 80 mA and exposure time of 3000 ms. The scan resolution was adjusted to 14 mm, voxel size to 27 mm, and, thresholding value to 1150. The reconstructed images were generated via the Microview v1.1.1 software (GE Medical Systems). The bone volume/tissue volume (BV/TV) of the upper and lower endplates of each IVD was calculated.

### 2.6. Histology

Upon fixation in 4% paraformaldehyde, the IVD tissue was decalcified, paraffin-embedded, and sliced into 7 *μ*m thick sagittal sections before staining with Hematoxylin & Eosin (H&E), Safranin O-Fast Green (SOFG), and Sirius red. The histological score was assessed by blinded independent researchers, as described previously [43, 44].

### 2.7. Scanning Electron Microscopy (SEM)

IVD morphology was assessed with a SEM (SEM; JSM-4800; Hitachi). In short, an IVD was cut along the coronal plane, fixed in 2.5% glutaraldehyde solution for 24 hours, and dried under partial vacuum. Then, the disc was sputter coated with gold and analyzed under accelerating voltage of 5-10 kV.

### 2.8. Transcriptome Sequencing (mRNA-seq) and Analysis

Total disc RNA was isolated and analyzed using Sinotech Genomics (Shanghai, China). Next, mRNA differential expression analysis was conducted with the R package edgeR. Differentially expressed RNAs with ∣log_2_(FC) |  value > 1 and *q* value < 0.05 were deemed significantly regulated and underwent additional analysis. Gene Ontology (GO) analysis for biological processes, cellular components, and molecular function as well as KEGG pathway analysis (Kyoto Encyclopedia of Genes and Genomes http://www.genome.ad.jp/kegg) was also performed via the enrich R package.

### 2.9. Real-Time PCR Assay

Total RNA was isolated from disc tissue samples (both NP and AF tissue) via the Total RNA Mini Kit (QIAGEN, Germany), following kit directions, and then converted to cDNA, prior to qPCR analysis via SYBR Green PCR Master Mix (TAKARA, Japan) and a Bio-Rad CFX96 PCR System (Bio-Rad, Australia). GAPDH was used as an internal reference. The primers were listed as follows: collagen II (F) 5′-CGAGGCAGACAGTACCTTGA-3′, (R) 5′-TGCTCTCGATCTGGTTGTTC-3′; Aggrecan (F) 5′-CTTCCCAACTATCCAGCCAT-3′, (R) 5′-TCACACCGATAGATCCCAGA-3′; MMP3 (F) 5′-GCTCATCCTACCCATTGCAT-3′, (R) 5′-GCTTCCCTGTCATCTTCAGC-3′; GAPDH (F) 5′-GGCACAGTCAAGGCTFAGAATG-3′, (R) 5′-GGTGGTGAAGACGCCAGTA-3′; Bmal1 (F) 5′-TCCGATGACGAACTGAAACAC-3′, (R) 5′-CTCGGTCACATCCTACGACAA-3′; Per2 (F) 5′-AGTGACGGGTCGAGCAAAG-3′, (R) 5′-TCATGTCGGGCTCTGGAATG-3′; Clock (F) 5′-GAACTTGGCGTTGAGGAGTCT-3′, (R) 5′-GTGATCGAACCTTTCCAGTGC-3′; Cry1 (F) 5′-GTCCAGCGACAGAGCAGTAAC-3′; (R) 5′-TACTGACTCTCCCACCAACTTCA-3′; Rev-ver*α* (F) 5′-GGAGGTGGTAGAATTTGCCAA-3′, (R) 5′-CCTTCACGTTGAACAATGACG-3′.

### 2.10. Western Blot

The IVD tissue was homogenized with a tissue homogenizer (Servicebio, China), before lysis with RIPA buffer (Beyotime) containing phosphatase and protease inhibitors for 10 minutes. The NP cells were directly lysed with RIPA lysate, and the resulting lysate underwent centrifugation at 12,000 g for 15 minutes. Protein quantification was done with a BCA analysis kit. The samples were next boiled for 15 minutes and the proteins were separated via SDS-PAGE, before transfer to PVDF membranes (Thermo Fisher Scientific). The membranes underwent blocking in 5% nonfat milk in TBS-Tween (TBST) at room temperature (RT) for 1 h, before overnight (ON) incubation with primary antibodies at 4°C. The primary antibodies used were anti-collagen II (1 : 1000, abcam) and MMP-3 (1 : 1000, abcam). After ON incubation, the membranes were rinsed three times in TBST and incubated with secondary HRP-linked antibodies for 1 h. Finally, the immunoreactive bands were assessed via chemiluminescent analysis (Bio-Rad).

### 2.11. Enzyme-Linked Immunosorbent Assay (ELISA)

To measure SOD and MDA, two common indicators of oxidative stress, IVD tissue was homogenized and centrifuged at 900 g at 4°C for 15 min, and supernatant was extracted. According to the manufacturer's instructions (Beyotime Biotechnology Co., LTD., Shanghai, China), the supernatant and standard substance were added into the colorimetric dish, and the OD value (530 nm) was recorded. SOD and MDA levels were calculated according to the standard curve.

### 2.12. Immunofluorescence

NP cell fixation was done in 4% paraformaldehyde for 10-15 minutes before permeabilizing with 0.2% Triton X-100 for 20 minutes. The cells underwent blocking in 10% normal goat serum at RT for 1 h, before incubation in primary antibodies against anti-collagen II (1 : 200) and MMP-3 (1 : 150) at 4°C. Next, cells were placed in secondary antibodies (FITC or Cy-3, 1 : 200) for 1 h at RT and finally incubated with DAPI for 10 min. Each step was followed by an additional 3 washing steps (15 min) with PBS. Cells were visualized under a fluorescence microscope, and fluorescence intensity quantification was done with ImageJ software.

### 2.13. Statistical Analysis

Data analyses were done with the SPSS 22.0 software (SPSS Inc., Chicago, IL). One way ANOVA or Student's *t*-test was used to identify significant differences (^∗^*P* < 0.05, ^∗∗^*P* < 0.01, and ^∗∗∗^*P* < 0.001). Data were expressed as mean with error bars denoting standard deviation.

## 3. Results

### 3.1. Effects of CR and PEMF Cotherapies on the Stability of IVDD

**Figure 1:**
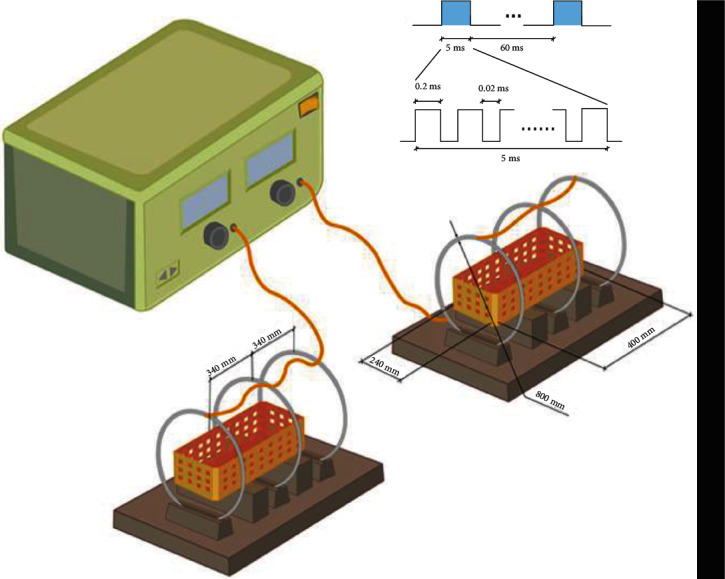
A schematic diagram of the PEMF exposure system and output waveform. The PEMF device composed of a signal generator and three Helmholtz coils with 800 mm coil diameters. The coils were placed coaxially 304 mm apart. The PEMF waveform comprised of a pulsed burst (burst width, 5 ms; pulse width, 0.2 ms; pulse wait, 0.02 ms; burst wait, 60 ms; pulse rise, 0.3 *μ*s; pulse fall, 2.0 *μ*s) repeated at 15 Hz.

**Figure 2:**
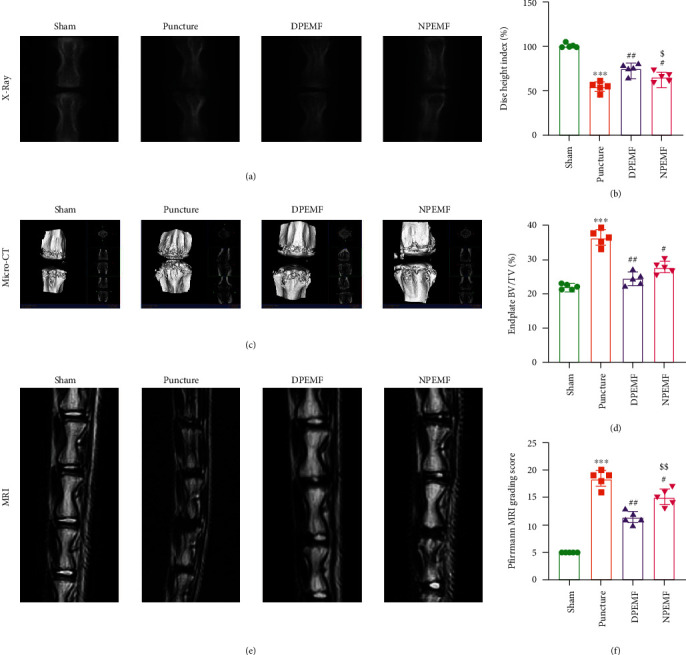
CR and PEMF cotherapies maintain the stability of IVDD. Imaging characteristics in each group after 8 weeks of treatment. (a, b) X-ray images of rat tail and the percent disc height index (%DHI), (c, d) micro-CT 3D reconstructions and endplate bone volume (BV/TV) measurements, and (e, f) MRI and IVDD grades (Pfirrmann grading score). ^∗∗∗^*P* < 0.001 vs. control rats; ^##^*P* < 0.01 vs. puncture rats; ^#^*P* < 0.05 vs. puncture rats; ^$$^*P* < 0.01 vs. DPEMF rats; ^$^*P* < 0.05 vs. DPEMF rats.

The rat tail needle puncture model representing IVDD was used to evaluate the therapeutic effect of CR and PEMF cotherapies on IVDD. As shown in Figure 1(a), loss of IVD height, scanned by X-ray, was observed in all puncture and treatment groups. In the DPEMF group, the disc height was markedly elevated than in the NPEMF rats (*P* < 0.05, Figures 2(a) and 2(b)), both of which were superior to the puncture rats (Figures 2(a) and 2(b)). In addition, the bone volumes (BV/TV) of the upper and lower endplate vertebral bodies were analyzed by micro-CT 3D reconstruction. Based on our results, both PEMF groups exhibited significantly lower BV/TV, compared to the puncture rats (*P* < 0.01, DPEMF *vs*. puncture; *P* < 0.05, NPEMF *vs*. puncture). Although there was a decreasing trend in the DPEMF group (Figures 2(c) and 2(d)), no obvious difference was seen between the DPEMF and NPEMF groups. Further MRI studies (Figure 2(e)) revealed that the IVD Pfirrmann grading score in the DPEMF rats was much lower, compared to the NPEMF (*P* < 0.01, Figures 2(e) and 2(f)) and puncture rats (*P* < 0.01 Figures 2(e) and 2(f)).

### 3.2. Effect of CR and PEMF Cotherapies on the Histology of the IVDD

**Figure 3:**
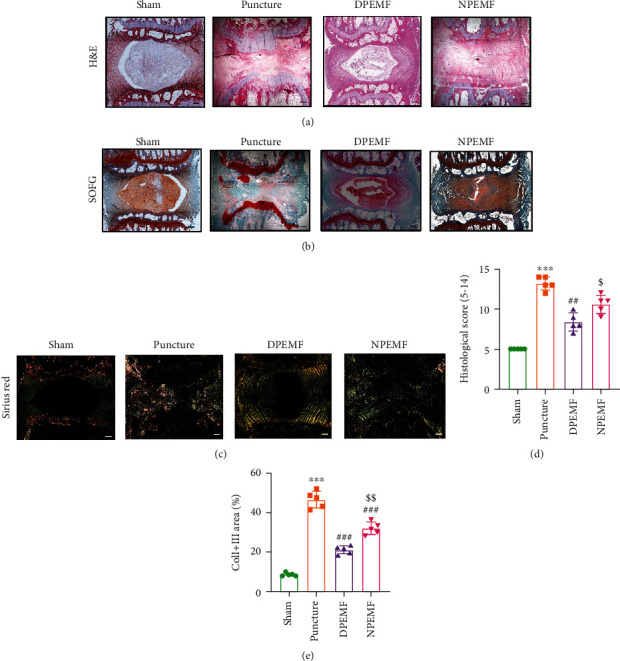
CR and PEMF cotherapies maintain the histology of punctured discs. Histological staining 8 weeks after puncture. (a, b, d) Representative images of Hematoxylin & Eosin (HE) and Safranin O-Fast Green (SOFG) stains and degenerative scoring based on staining (scale bar = 500 *μ*m). (c) Polarized Sirius red staining showing distribution and orientation of the type I and type III collagen in AF (scale bar = 500 *μ*m). (e) Analysis of type I and type III collagen area, based on the Sirius red staining. ^∗∗∗^*P* < 0.001 vs. control rats; ^###^*P* < 0.001 vs. puncture rats; ^##^*P* < 0.01 vs. puncture rats; ^$$^*P* < 0.01 vs. DPEMF rats; ^$^*P* < 0.05 vs. DPEMF rats.

Histological examinations were performed to evaluate the morphological changes after PEMF treatment. H&E (Figure 3(a)) and SOFG (Figure 3(b)) staining showed normal morphology and structure in the sham group, including proteoglycan-rich NP tissue, well-arranged AF, complete cartilage endplates, and clear AF-NP boundary. In the puncture group, a small number of irregularly shaped NP cells were seen, with fiber lamellae protruded inwardly in the AF, while the endplate was partially disrupted, and the AF-NP boundary was unclear. In the DPEMF treatment group, parts of the NP tissue were restored, and AF lamellae protruded inward in a moderately compensatory manner. Conversely, in the NPEMF treatment group, a small part of the NP tissue was left, and the AF structure appeared less disordered than in the puncture group. The histological score was highest in the puncture group, followed by NPEMF group, and then the DPEMF and sham groups (Figure 3(d)), indicating that the DPEMF was superior to NPEMF in maintaining IVDD histology.

**Figure 4:**
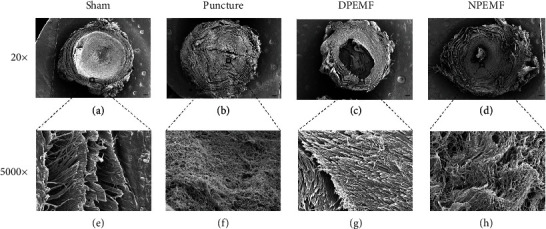
CR and PEMF cotherapies reduce morphological changes in puncture discs. Surface morphology of discs in each group under scanning electron microscopy (SEM). (a–d) Representative IVD coronal morphology, under 20x magnification (scale bar = 500 *μ*m). (e–h) Representative images of AF-NP boundary, under 5000x magnification (scale bar = 2 *μ*m).

Sirius red staining (Figure 3(c)) under polarized light showed that type I and type III collagens were orderly arranged and NP (mainly contain type II collagens) in the center were totally black in the IVDs of the sham group. During IVDD, however, the type I and type III collagens gradually replaced type II collagen. Moreover, the percentage of type I and type III collagen areas in the DPEMF and NPEMF rats was markedly lower, compared to the puncture-only rats (*P* < 0.001, DPEMF rats; *P* < 0.001, NPEMF rats). In addition, the percentage of type I and type III collagen area was remarkably elevated in the NPEMF rats, relative to the DPEMF rats (*P* < 0.01, Figure 3(e)). Furthermore, SEM (Figures 4(a)–4(h)) was used to observe the structural changes within the fibers of the AF-NP boundary. We revealed, using high magnification (5000x), that the fibers in the AF-NP boundary were densely and orderly arranged in sham rats. In puncture rats, however, the fibrous structure was sparse and honeycombed. Both PEMF groups showed less disorganization than the puncture group, and morphological alterations in the DPEMF rats were more organized, compared to the NPEMF rats. Taken together, these findings confirm the beneficial effect of PEMF on IVDD, with a particularly profound effect achieved by DPEMF.

### 3.3. Effect of CR and PEMF Cotherapies on the Expression of ECM-Related Genes

**Figure 5:**
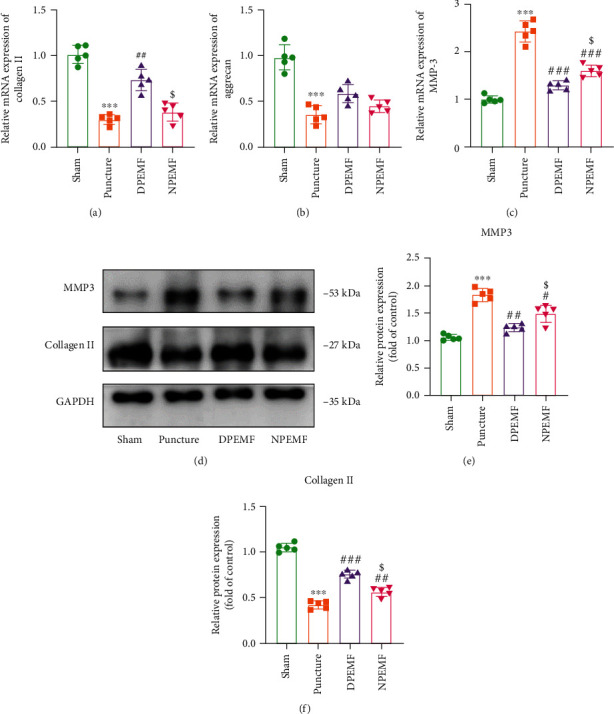
Effects of CR and PEMF cotherapies on extracellular matrix (ECM) maintenance. (a–c) qRT-PCR analysis of COL2, Aggrecan, and MMP-3 (*n* = 5). (d–f) Western blot and semiquantitative analysis of COL2 and MMP-3 levels (*n* = 5). ^∗∗∗^*P* < 0.001 vs. control rats; ^###^*P* < 0.001 vs. puncture rats; ^##^*P* < 0.01 vs. puncture rats; ^$^*P* < 0.05 vs. DPEMF rats.

The mRNA and protein expressions of collagen II, Aggrecan, and MMP-3 were examined via RT-PCR and Western blotting at 8 weeks after surgery. As depicted in Figure 5(a), the mRNA levels of collagen II in the DPEMF rats were markedly elevated, compared to the puncture and NPEMF rats, respectively (*P* < 0.01, DPEMF *vs.* puncture group; *P* < 0.05, DPEMF *vs.* NPEMF group). The mRNA expression of Aggrecan decreased significantly in the puncture only group, with respect to the sham group. PEMF treatment slightly upregulated Aggrecan expression, but no significance was achieved (Figure 5(b)). In addition, the mRNA level of MMP-3 was highest in the puncture group, followed by the NPEMF group and then the DPEMF and sham groups (Figure 5(c); *P* < 0.001, DPEMF *vs.* puncture; *P* < 0.05, DPEMF *vs.* NPEMF). The MMP-3 and collagen II protein expressions were also examined in this study. The MMP-3 protein expression in the PEMF groups was drastically reduced, compared to that in the puncture group (Figure 5(e); *P* < 0.01, DPEMF *vs.* puncture; *P* < 0.05, NPEMF *vs.* puncture), with a much lower level in the DPEMF group (Figure 5(e); *P* < 0.05, DPEMF *vs.* NPEMF). However, the collagen II protein expression in the PEMF groups was markedly elevated, relative to the puncture group (Figure 5(f); *P* < 0.001, DPEMF *vs.* puncture; *P* < 0.01, NPEMF *vs.* puncture), with a much higher level in the DPEMF group (Figure 5(f); *P* < 0.05, DPEMF *vs.* NPEMF). These collective findings indicate that PEMF strongly modulates the expression of ECM-related genes, and DPEMF has a more pronounced effect, relative to NPEMF.

### 3.4. Effect of Circadian Cycle Response on the PEMF-Mediated Stimulation in NP Cells

**Figure 6:**
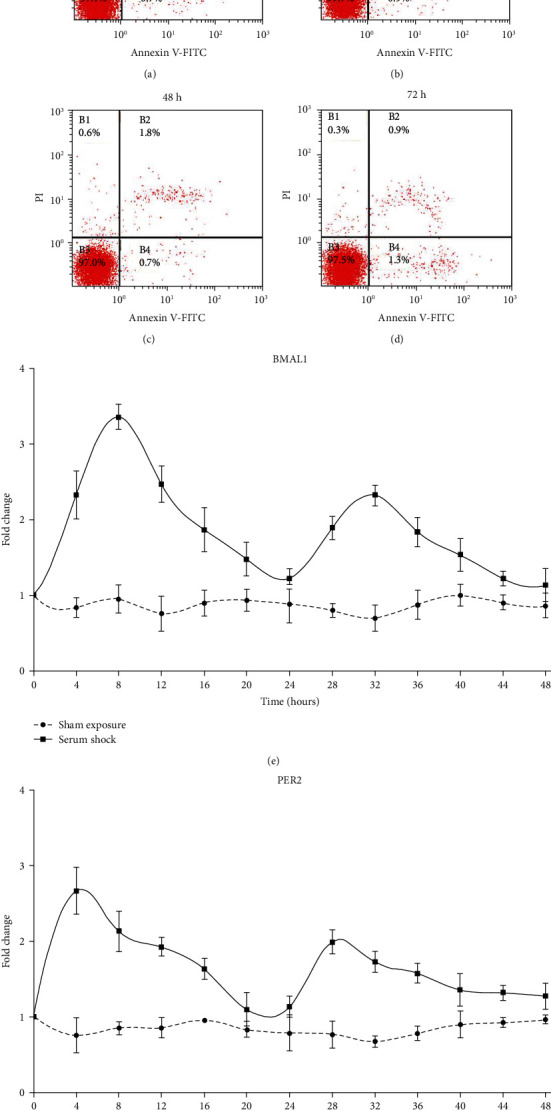
(a–d) The apoptosis ratio of NP cells at time points 24 h, 48 h, and 72 h after 4 h of PEMF treatment. The 0 h time point indicates no PEMF treatment. (e, f) mRNA expression of circadian genes Bmal1 and Per2 in NP cells during 48 h with or without serum shock. Serum shock was provided at time point 0 h. SS: serum shock.

**Figure 7:**
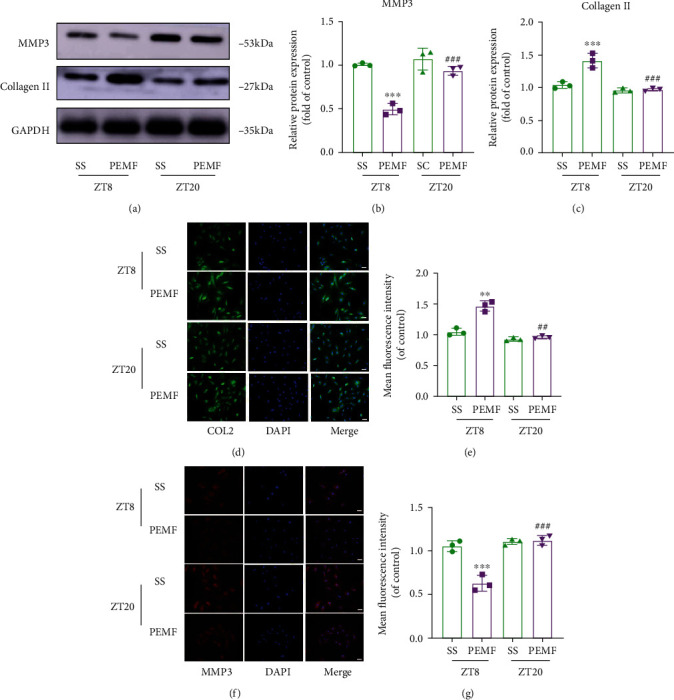
Effects of PEMF on collagen II (COL2) and MMP-3 expression in NP cells. (a–c) Western blot and semiquantitative analysis of COL2 and MMP-3 expressions (*n* = 3). (d–g) Representative images of immunofluorescence and semiquantitative analysis of COL2 and MMP-3 fluorescence intensity in NP cells, imaged by fluorescence microscopy (scale bar = 100 *μ*m). ^∗∗∗^*P* < 0.001 vs. ZT8 control rats; ^∗∗^*P* < 0.01 vs. ZT8 control rats; ^###^*P* < 0.001 vs. ZT20 control rats; ^##^*P* < 0.01 vs. ZT20 control rats.

Since NP cells play a crucial role in IVDD, we next explored the potential impact of PEMF on NP cells. The apoptosis rate was detected via flow cytometry at 24 h, 48 h, and 72 h after 4 h PEMF treatment. Based on our analysis, the apoptosis rate of PEMF-treated NP cells at 24 h, 48 h, and 72 h time points was similar to that of the cells that received no PEMF stimulation (Figures 6(a)–6(d)), indicating that PEMF stimulation is safe for NP cells. Next, we performed serum shock to imitate the circadian gene oscillation model *in vitro*. As expected, we observed circadian gene oscillation in the NP cells that received serum shock and no oscillation in the untreated cells (Figures 6(e) and 6(f)). Next, PEMF was applied to the NP cells at the Zeitgeber Times ZT8 and ZT20 (ZT0 was the start time of serum shock), which represented two typical time points that produced peaks and troughs in the cycle of the circadian clock gene oscillation. As shown in Figure 7, PEMF at different time points showed remarkably different results. PEMF at ZT8 demonstrated a strong protective effect, as evidenced by the elevated levels of ECM component protein COL2 and lower levels of ECM degeneration-related protein MMP-3, demonstrated via WB and immunofluorescence (Figures 7(a)–7(g)). However, PEMF at ZT20 demonstrated no difference in ECM-related gene expression, compared to the serum shock group. The above results indicate that the PEMF treatment displays a diurnal variation in NP cells; thus, clock genes are involved in the chronotherapy-mediation regulation of PEMF treatment.

### 3.5. Transcriptome Sequencing Analysis Revealed the Underlying Mechanism in the Differential Regulation of Chronotherapy on PEMF Treatment

**Figure 8:**
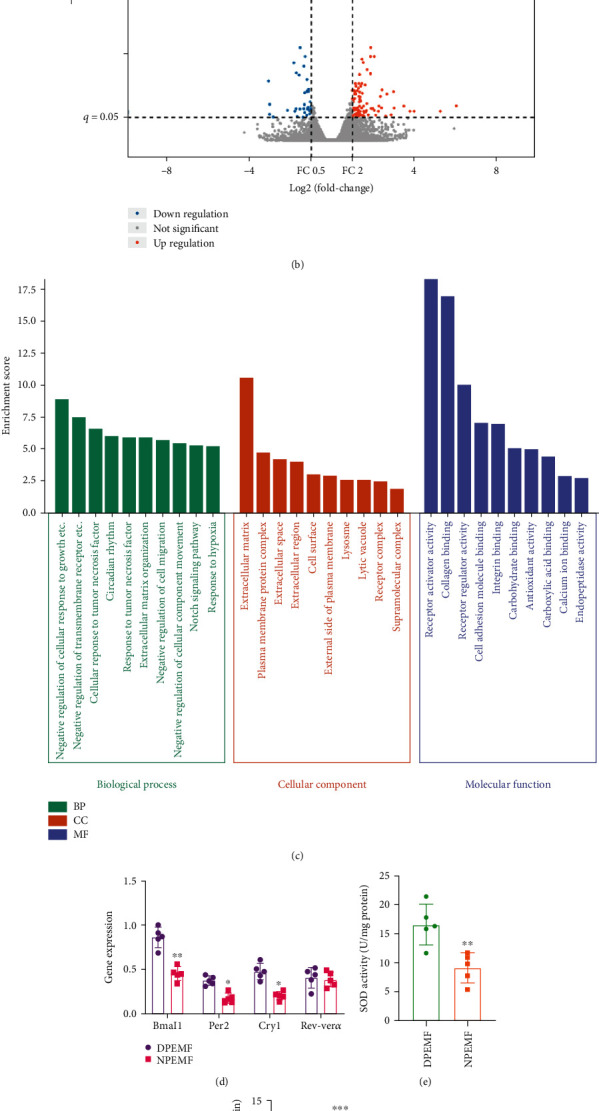
IVD transcriptome profiles in the DPEMF and NPEMF groups. (a) IVD DEG heat map between the DPEMF and NPEMF groups. (b) IVD DEG volcano map comparing the DPEMF vs. NPEMF groups. Red dots represent 71 markedly elevated DEGs; blue dots represent 36 markedly reduced DEGs; and gray dots represent no change. (c) GO terms. (d) IVD mRNA expressions in both DPEMF and NPEMF groups, using RT-PCR analysis (*n* = 5 per group). ^∗∗^*P* < 0.01 vs. DPEMF rats; ^∗^*P* < 0.05 vs. DPEMF rats. (e, f) SOD activity and MDA content in DPEMF and NPEMF groups. (g) KEGG pathway.

Discs in the DPEMF and NPEMF rats were next subjected to transcriptome sequencing to identify the potential mechanism underlying the differential effect of CR and PEMF cotherapies. Based on the volcano maps, 71 differentially expressed genes (DEGs) were upregulated and 36 were downregulated between the DPEMF and NPEMF groups (Figure 8(b)). The DEG heat map between DPEMF and NPEMF groups is presented in Figure 8(a). To better understand the potential DEG functions between the two treatment groups, GO enrichment and KEGG analysis were performed to unveil mechanism(s) regulated by chronotherapy and PEMF. The top 10 GO term enrichments between the DPEMF and NPEMF groups in different categories are illustrated in Figure 8(c). Based on our data, different cellular components, biological processes, and molecular functions were enriched in the DPEMF and NPEMF groups. For instance, in molecular functions, differential genes of antioxidant activity were enriched, which indicated differences in antioxidant stress between DPEMF and NPEMF groups. Moreover, in vivo results confirmed that DPEMF upregulated SOD activity compared with NPEMF and displayed a lower MDA level. Circadian rhythm-related genes were also enriched, suggesting a possible role in PEMF treatment. The mRNA levels of four key clock genes Bmal1, Cry1, Per2, and Rev-ver*α* were verified by RT-qPCR. The expression of clock genes Bmal1, Cry1, and Per2 but not Rev-ver*α* was significantly elevated in the DPEMF rats, compared to the NPEMF rats (*P* < 0.01; *P* < 0.05; *P* < 0.05). Further KEGG pathway analysis unveiled that DEGs between DPEMF and NPEMF were mainly involved in the PI3K-AKT signaling pathway, ECM-receptor interaction, cGMP-PKG signaling pathway. Collectively, the above results indicate that circadian rhythm-related genes in IVD tissues may be a potential factor influencing the differential therapeutics of PEMF in daytime and nighttime, and the PI3K-Akt signaling pathway may be the key signaling leading to the treatment differences.

## 4. Discussion

In this study, we systematically assessed the chronotherapy-mediated regulation of PEMF treatment of IVDD induced by tail disc puncture in rats for 8 weeks. In our in vivo study, we revealed that the degeneration progress of both PEMF rats (DPEMF and NPEMF rats) were drastically lower, compared to the puncture-only rats, which indicated that PEMF stimulation can, in fact, promote functional recovery after IVDD. Furthermore, the DPEMF group exhibited significantly enhanced performance, compared to the NPEMF group, as evidenced by imaging analysis, histomorphology assessment, and ECM molecule expression. These results suggest that CR may affect the therapeutic effect of PEMF on IVDD. In our *in vitro* study, we employed the serum shock model to induce oscillating circadian clock gene expression in NP cells, thus simulating the biological rhythm *in vivo*. We demonstrated that PEMF administration during a period of high circadian clock gene expression (equivalent to the treatment period in rats during the day) resulted in a better outcome of minimizing degeneration than during the time when the circadian clock gene expression was low, indicating that clock genes are involved in the therapeutic effect of PEMF on IVD. Collectively, these results suggest that CR plays a crucial role in treating IVDD with PEMF.

It was reported earlier that PEMF exerts therapeutic effects via regulation of metabolic pathways. This is achieved by modulating cell proliferation, differentiation, and maturation, especially in the treatment of musculoskeletal diseases [45–47]. In clinical studies, PEMF was also reported to improve therapeutic efficacy of multiple bone diseases, including cartilage defects, tendon repair, osteoarthritis, and osteoporosis [48–50]. Thus far, several studies have shown that PEMF can relieve acute inflammatory responses in a short term, seven-day treatment in a rat model of IVD degeneration, while the long-term effects of PEMF have not been studied [11]. In addition, recent studies have yielded inconsistent results in *in vivo* and *in vitro* studies, suggesting that there may be other key factors, besides PEMF parameters and duration, influencing the therapeutic effect of PEMF [11, 13, 14]. In our previous studies, we have identified a diurnal variation in the efficiency of PEMF on osteoporosis and peripheral nerve regeneration, which inspired us that circadian rhythm might be a potential key factor affecting the therapeutic efficacy of PEMF on disc degeneration [30, 31, 34]. Using the CR principle to guide the timing of treatments can not only improve the efficacy of treatments but also reduce the side effects, which has been shown to be effective in the treatment for several diseases, such as rheumatoid arthritis and cancer [51, 52]. A recent study has demonstrated that LBP symptoms by IVD degeneration show a diurnal variation [53]. However, such chronotherapy treatment principle has not been tested on IVDD.

To systematically assess the synergy between PEMF and CR in treating IVDD, we compared the effect of CR and PMEF cotherapies on IVDD. It is well known that IVDD clinical diagnosis is highly dependent on imaging, such as X-ray, CT, and MRI. Moreover, MRI is the current gold standard for evaluating IVDD. Using imaging analysis, we revealed that PEMF is highly capable of alleviating IVDD, as evidenced by increased IVD height, EP bone volume, and improved MRI signal. In addition, the above outcomes in the DPEMF rats were remarkably enhanced, compared to the NPEMF rats. Additionally, we assessed the effects of CR and PEMF cotherapies on histomorphological changes. We demonstrated that, in the PEMF rats, the histomorphology of NP, AF, and EP was markedly better, relative to the puncture rats. In the DPEMF group, NP was partially preserved, with large amounts of visible regular AF and highly organized EP structure. In the IVDD process, however, the degradation process deteriorates the NP tissue and decreases its ability to maintain significant hydrostatic pressure, which eventually fails to prevent the immersion of the inner AF. Here, we used SEM to observe the structural alterations in the AF-NP boundary during IVDD. Interestingly, we observed the fiber direction was more regularly organized in the PEMF groups than in the puncture-only group. In addition, the AF-NP boundary structure in the DPEMF rats was more organized, compared to the NPEMF rats. In the IVDD process, the degradation process was accompanied by an imbalance in ECM generation and degradation, which was characterized by decreased proteoglycan and type II collagen and increased matrix metalloproteinase. After PEMF treatment, the imbalance caused ECM generation, as evidenced by increased proteoglycan and type II collagen and decreased matrix metalloproteinase. In addition, the DPEMF was more effective than the NPEMF in promoting ECM generation and in alleviating ECM degradation. These results indicate that the therapeutic outcome of PEMF in IVDD is partially dependent on CR, with a better outcome occurring in the DPEMF-treated rats. However, the mechanism underlying the chronotherapy-mediated regulation of PEMF-based IVDD treatment is still unknown.

CR in the central brain and peripheral tissues together affects IVD physiology [29]. CR in central originates in the suprachiasmatic nucleus (SCN) of the hypothalamus and coordinates neuronal and hormonal signals to keep peripheral tissues in sync with external environment [54]. CR in peripheral tissues depends on core molecular clock genes, such as transcriptional activator (Clock, Bmal1) and repressors (Per1/2 and Cry1/2) [27]. These genes generate rhythmic oscillations to drive other gene transcription and translation for maintenance tissue-specific rhythms. Therefore, the enhanced therapeutic effects associated with DPEMF radiation may probably be due to the direct effect of rhythmic states of disc cells and, maybe, some indirect effect of diurnal hormone signals. A recent study has demonstrated the existence of clock genes within IVD that controls key aspects of physiology and pathophysiology by rhythmically regulating the expression of parts of the IVD transcriptome, enabling cells to respond to biomechanical and chemical changes [27], whereas disruption of clock genes accelerates tissue aging and makes IVD more susceptible to degeneration.CR strongly regulates the therapeutic effect of PEMF on IVDD in rats, which is supported by both our *in vivo* and *in vitro* data. It is well recognized that PEMF affects the endogenous clock that controls the sleep-wake cycle of animals, suggesting that PEMF stimulation can alter the natural rhythm of animals [55]. Our transcriptome sequencing results concurred with this in that PEMF stimulation increased the expression of circadian genes in IVD tissues and its function was enhanced in the presence of upregulated clock genes. This strongly indicates that PEMF enhances its function partially by upregulating the expression of clock genes in tissues. Hence, it would be interesting to further examine how CR regulates PEMF modulation of the biological clock genes and its role in IVDD, which is also a limitation of our study. On the other hand, the better effect of DPEMF may also be mediated indirectly by different hormone levels. Emerging evidences, in recent years, recognized that melatonin is highly beneficial for IVDD [56–59]. In addition, the melatonin dose must be high enough to elicit a beneficial effect on IVDD. Other hormones such as parathyroid hormone (PTH) also show diurnal alterations in serum concentration and are able to reset the mouse cartilage clock *in vitro*. NP cells express type 1 PTH receptors and respond to PTH stimulation, indicating that these cells may be sensitive to circadian entrainment by PTH [60–62]. It would be interesting to explore how CR regulates PEMF-mediated regulation of hormone secretion and whether this influences IVDD in future studies.

In short, in rats, the DPEMF stimulation is more effective than the NPEMF in alleviating IVDD. However, given that rats and humans exhibit opposite sleep-wake cycles and circadian activity and that there is an offset of about 12 hours in humans, relative to rats, we therefore speculate that in humans the NPEMF therapy may be more conducive to alleviating IVDD than the DPEMF therapy. However, the exact role of PEMF in human NP will be studied in our future research. Moreover, we hope to translate our conclusions into a clinical setting to further optimize IVDD treatment outcomes, by combining CR and PEMF, in an attempt to improve patient rehabilitation programs. For instance, patients can use portable electromagnetic devices for PEMF radiation at night at home, instead of receiving it during the day at a health rehabilitation center. As chronotherapy has a small cost to healthcare services and patients, as well as has the potential to bring significant benefits, future therapeutic interventions for IVDD may also benefit from such principles.

## Data Availability

The data used to support the findings of this study are available from the corresponding authors upon request.
